# Heterogeneity of cost estimates in health economic evaluation research. A systematic review of stress urinary incontinence studies

**DOI:** 10.1007/s00192-018-3814-0

**Published:** 2019-02-04

**Authors:** Sandra Zwolsman, Arnoud Kastelein, Joost Daams, Jan-Paul Roovers, B. C. Opmeer, On behalf of the WOMEN-UP Consortium: http://www.women-up.eu/consortium/

**Affiliations:** 1 Department of Gynaecology and Obstetrics, Amsterdam UMC, Meibergdreef 9, 1105AZ Amsterdam, The Netherlands; 2 Gynaecology and Obstetrics, Amsterdam UMC, Room H4-232, Postbox 22770, 1100 DE Amsterdam, the Netherlands; 3 Medical Library, Amsterdam UMC, Meibergdreef 9, 1105AZ Amsterdam, the Netherlands; 4 Clinical Research Unit, Amsterdam UMC, Meibergdreef 9, 1105AZ Amsterdam, the Netherlands

**Keywords:** Health care costs, Health economic evaluation research, Health technology, Multinational, Standardized unit costs, Transferability

## Abstract

**Introduction and hypothesis:**

There is increased demand for an international overview of cost estimates and insight into the variation affecting these estimates. Understanding of these costs is useful for cost-effectiveness analysis (CEA) research into new treatment modalities and for clinical guideline development.

**Methods:**

A systematic search was conducted in Ovid MEDLINE & other non-indexed materials and Ovid Embase for articles published between 1995 and 2017. The National Health Service Economic Evaluation Database (NHS-EED) filter and the McMaster sensitive therapy filter were combined with a bespoke search strategy for stress urinary incontinence (SUI). We extracted unit cost estimates, assessed variability and methodology, and determined transferability.

**Results:**

We included 37 studies in this review. Four hundred and eighty-two cost estimates from 13 countries worldwide were extracted. Descriptive analysis shows that hospital stay in gynecology ranged between €82 and €1,292 per day. Costs of gynecological consultation range from €30 in France to €158 in Sweden. In the UK, costs are estimated at €228 per hour. Costs of a tension-free vaginal tape (TVT) device range from €431 in Finland to €994 in Canada. TVT surgery per minute costs €25 in France and €82 in Sweden. Total costs of TVT range from €1,224 in Ireland to €5,809 for inpatient care in France. Variation was explored.

**Conclusions:**

Heterogeneity was observed in cost estimates for all units at all levels of health care. CEAs of SUI interventions cannot be interpreted without bias when the base of these analyses—namely costs—cannot be compared and generalized.

## Introduction

Urinary incontinence is a common condition in women with annual costs of nearly 10 billion euros in both direct and indirect costs in Europe [1]. Most of these women suffer from stress urinary incontinence (SUI) [2, 3]. The costs for diagnosis, treatment, and follow-up of patients with SUI differ among countries. Insight into these costs is useful for cost-effectiveness research into new treatment modalities and for clinical guideline development. A clear international overview of these cost estimates is currently lacking. Therefore, information on costs should be gathered from health economic research data. Economic data from cost-effectiveness studies shows great variability [4], which prevents the reliable use of these data.

Although variation among countries and hospitals with respect to relative prices is expected, there are other factors that cause variation. Units of resource use are often not clearly described and defined, introducing great variability in cost estimates. Moreover, lack of transparency in the costing methodology and procedures used in health economic evaluation research hamper insight into the composition of costs. In the field of female pelvic floor medicine and reconstructive surgery, Rawlings and Zimmern demonstrated that cost estimates for unit measures vary considerably. Costs were commonly not itemized, not all relevant costs were reported, and indirect costs were inconsistent and not always considered [5]. Furthermore, de Soarez et al. demonstrated that the methodology and quality of health economic evaluations is often inadequate, and that costs cannot be transferred internationally [6]. Other authors showed considerable heterogeneity in cost estimations for the prevention, detection, and treatment of various diseases [7, 8].

There is a demand for an international overview of cost estimates for SUI and insight into the variation affecting these estimates [9]. As outlined above, cost estimates generated by health economic evaluation research cannot be used without caution. This review is aimed at providing an overview of cost estimates for different components in the diagnosis, treatment, and follow-up of SUI. Furthermore, we aimed to assess variation in cost estimates for SUI, and explore factors causing this variation. We also offer a preliminary recommendation for the incorporation of economic data from economic research at a national level into multinational cost-effectiveness analysis.

This overview facilitates researchers performing cost-effectiveness research and clinical guideline development in the field of SUI.

## Materials and methods

We conducted a systematic review of health economic evaluation studies that addressed costs reported in comparative analyses of diagnostic procedures or treatment modalities for women with SUI.

### Search

The search strategy was developed in collaboration with a medical librarian (JD). A scoping search based on reference checking and citation analysis (“cited by” and citing articles) was conducted. Results from this search were used to derive key concepts and to identify relevant articles that had to be retrieved by the systematic search.

The systematic search was conducted in July 2017 in the Ovid MEDLINE & other non-indexed materials and Ovid Embase bibliographic databases. The National Health Service Economic Evaluation Database (NHS-EED) filter for identifying economic evaluations and the McMaster sensitive therapy filter were combined with a search strategy for SUI. In addition, the WHO ICTRP search portal was searched to identify relevant trials. The retrieved trial numbers were included in the systematic search strategy. No additional limits were applied. Full details of the search strategy can be found in the appendix.

### Selection of articles

References for studies that were identified using the search strategy were imported in Covidence. BCO, MD, and SZ separately and independently screened titles and abstracts for relevance using the inclusion and exclusion criteria stated below.

### Inclusion and exclusion criteria

We included studies on adult women with SUI that complied with the following criteria:

1. Screening or diagnostic testing, conservative interventions (i.e., medication or pelvic floor muscle training) or surgery, or use of medical devices or other management of SUI
2. Comparative studies (at least two interventions)
3. Full economic evaluations (taking into account both effectiveness, i.e., health outcomes and/or quality of life, and costs)
4. Primary study design, either empirical (clinical cohort or trial) or model-based (for instance, decision tree or Markov model)
5. Published in any scientific journal from peer-reviewed journals between January 1995 and July 2017

Exclusion criteria were:

1. Studies performed in elderly patients or in nursing homes
2. Opinion pieces, short communications and conference proceedings
3. Cost-of-illness studies
4. Cost-consequence analyses
5. Reviews

### Selection of full-text articles

After screening titles and abstracts, full article texts were evaluated for relevance. In case of discrepancies, BCO and SEZ discussed the contents of the article until consensus was reached. If consensus was not reached, the article was presented to a third objective reviewer. All full-text articles were retrieved and used for this review.

### Data extraction

A data extraction sheet was developed to extract key bibliographic characteristics and relevant data for health economic evaluation studies [10]. We extracted general data (author, year, country, setting, type of intervention evaluated, costing characteristics, etc.), and type of economic analysis.

All reported cost estimates and associated units costs were extracted and categorized according to the following rubrics: admissions, adverse events, consultant, diagnostics, incontinence material, laundry, medical equipment, medication, procedure, productivity, and travel [11]. Within these rubrics, further classification was based on the level of aggregation, to allow meaningful comparisons. Costs were extracted and categorized according to the level of aggregation. A high level of aggregation incorporated surgical procedures that included all costs related to staff time, operating theater, surgical disposables, and hospital stay for recovery. A low level of aggregation included hourly costs of a gynecologist, surgeon or nurse; purchase costs of incontinence material, etc. We therefore classified and presented the results for various cost estimates by classifying them as having a high, medium or low level of aggregation, and medium- or low-level cost estimates were organized and reported in the following rubrics: admissions to health care institutions, diagnostic procedures, health care providers, surgical procedures, or materials. For instance, within the rubric “consultations,” the categories are general practitioner (GP), physiotherapist, surgeon, anesthesiologist, nurse visits, and hourly wages for GPs, surgeons, nurses, etc.

### Indexation and country adjustment of cost estimates and unit costs

To allow valid and meaningful comparisons of unit cost estimates within and between countries, a common price level is required for each country in addition to the reported price year, so unit costs can be converted. Reported cost estimates were converted to 2017 Euros at the Dutch price level, adjusted for price year using the consumer price index for each country of July of the reported year and the year 2017, and for purchasing power using OECD comparative purchasing power parities [12, 13]. If no price year had been reported, July of the year before the publication year was assumed to be the price year. When the price year ranged over 2 years, which we have seen in the literature search, for instance 2000–2001, July of the first year was assumed to be the price year. In one article mentioning unforeseen hospitalization costs as a percentage of the total population, costs per day for one person were recalculated.

### Determining transferability of costs

Transferability of costs was determined using the criteria of Fukuda et al. These authors describe four levels of transferability, depending on the extent to which components of costs and data for costs are reported. The method of calculating unit costs is also taken into account [8].

The following levels of transferability are taken into account, as cited from Fukuda et al. [8]:

A All components of costs were described and data for both quantity and unit price of resources were reported for each component
B All components of costs were described and data for costs in each component were reported. This included studies that used graphical presentations of the aforementioned data
C All components of costs were described, but data for costs in each component were not reported
D Only the scope of costing was described, but the components of costs were not described

The following standards were used to apply the abovementioned categories to our data:

A Qualitative economic data of quantities and unit prices for each component for which costs were described
B Economic data of quantities and unit prices where costs were weighted or averaged
C Data in categories where no separate components or units are described
D Overall cost data (=costs for a procedure without further specification of components)

### Determining the quality of costing methodology

Costing methodologies are categorized according to the following quality criteria, also cited from Fukuda [8]:

I Micro costing or quasi-micro costing
II Use of relative value units
III Use of ratio of costs to charges
IV Unmodified charge data
V Unknown

### Reporting of cost estimates and unit costs

Adjusted cost estimates of hospital admissions (=cost of a hospital bed per day) were presented graphically to illustrate the variability in reported prices for the same cost estimate within and between countries (see Fig. 2). Costs were presented as provided in the literature (cost per day) or costs per 8 h if hourly wages or wages for multiple days were presented. In addition, for the most common unit of health care use in the treatment of SUI—hospital admission (day admission for inpatient or outpatient procedure)—more detailed reporting of (variation in) unit costs was provided, including the source and variation of the cost estimates.

An overview of costs for tension-free vaginal tape (TVT) and urodynamic testing was also given.

## Results

### Selection of articles

**Fig. 1:**
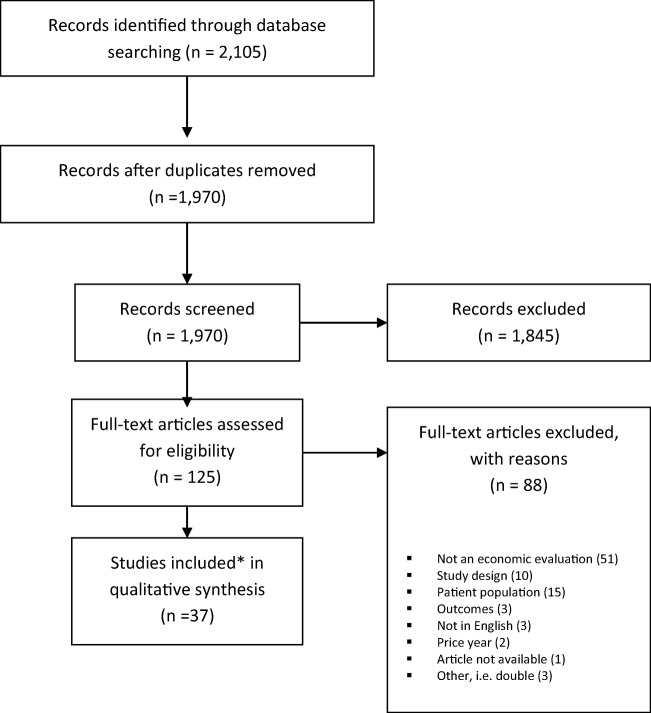
Flowchart. *Asterisk* studies included in this review come from the following countries: Australia, Bosnia-Herzegovina, China, Canada, Egypt, Finland, France, Ireland, Netherlands, Spain, Sweden, United Kingdom, United States

Of the 1,980 articles identified using our search strategy (Tables 1, 2), a total of 37 articles were selected and included in this review (Fig. 1). Reasons for exclusion were, for example, not an economic evaluation, but cost outcome, unsuitable study design (study protocol), unsuitable patient population (no SUI, but mixed UI, gender), and price year before 1995 (could not be adjusted). As we selected articles using the online platform Covidence, agreement could not be calculated. A summary of all the studies included is presented in Table 3.

**Table 1:** Search strategy in MEDLINE

	Ovid MEDLINE® Epub Ahead of Print, In-Process & Other Non-Indexed Citations, Ovid MEDLINE® Daily and Ovid MEDLINE® < 1946 to Present > Search date: 20 July 2017 (initial search: 25 November 2015)	
Number	Searches	Results
1	Economics/or exp. “costs and cost analysis”/or Economics, Dental/or exp. economics, hospital/or Economics, Medical/or Economics, Nursing/or Economics, Pharmaceutical/	261,337
2	(economic$ or cost or costs or costly or costing or price or prices or pricing or pharmacoeconomic$ or (expenditure$ not energy) or “value for money” or budget$).ab,ti.	666,619
3	1 or 2	782,450
4	(((energy or oxygen) adj cost) or (metabolic adj cost) or ((energy or oxygen) adj expenditure)).ti,ab.	25,882
5	3 not 4	776,532
6	(letter or editorial or historical article).pt.	1,756,196
7	5 not 6	743,463
8	exp animals/not humans	4,442,323
9	7 not 8	698,668
10	(bmj or “cochrane database of systematic reviews” or “health technology assessment winchester england”).jn.	87,780
11	9 not 10 [NHS-EED filter MEDLINE, consulted 20,151,104]	693,011
12	clinical trial.mp.	672,213
13	clinical trial.pt.	523,453
14	random:.mp. or tu.xs.	5,075,317
15	or/12–14 [McMaster sensitive therapy filter]	5,242,693
16	exp enuresis/or exp. urinary incontinence/	34,569
17	(((urin* or bladder) adj3 incontin*) or encopresis or enures* or ((urin* or bladder) adj2 control) or (urin* adj2 (leak* or hold*)) or icq or interstim).ab,kf,ti.	34,578
18	16 or 17 [urinary incontinence]	48,646
19	11 and 15 and 18	615
20	(NCT02316275 or ISRCTN57746448 or NTR3783 or NCT01239836 or NTR1871 or NCT00814749 or NTR1248 or NCT00611026 or NTR1181 or NCT00509730 or NCT00498888 or NCT00444925 or NCT00425100 or ACTRN12605000755639 or NCT00200031 or ISRCTN97769568).ab,kf,ti.	6
21	(MiniMo or VUSIS or “Value of Urodynamics Prior to Stress Incontinence Surgery”).ab,kf,ti.	167
22	20 or 21 [relevant urinary incontinence trials]	171
23	19 or 22	784
24	remove duplicates from 23	738

**Table 2:** Search strategy in Embase

	Embase Classic + Embase < 1947 to 2017 July 19 > ‘. Ovid interface. Search date: 20 July 2017 (initial search: 25 November 2015)	
Number	Searches	Results
1	Health Economics/or exp. Economic Evaluation/or exp. Health Care Cost/ or pharmacoeconomics/	469,173
2	(econom$ or cost or costs or costly or costing or price or prices or pricing or pharmacoeconomic$ or (expenditure$ not energy) or (value adj2 money) or budget$).ab,ti.	876,670
3	1 or 2	1,084,623
4	(letter or editorial or note).pt.	2,207,777
5	3 not 4	995,541
6	((metabolic adj cost) or ((energy or oxygen) adj cost) or ((energy or oxygen) adj expenditure)).ti,ab.	31,413
7	5 not 6	988,967
8	animal/or exp. animal experiment/or nonhuman/	7,360,425
9	(rat or rats or mouse or mice or hamster or hamsters or animal or animals or dog or dogs or cat or cats or bovine or sheep).ti,ab,sh.	5,915,466
10	8 or 9	8,581,286
11	exp human/or human experiment/	18,874,775
12	10 not (10 and 11)	6,515,262
13	7 not 12	903,173
14	(0959–8146 or 1469-493X or 1366–5278).is.	76,948
15	1756–1833.en.	24,248
16	14 or 15	94,260
17	13 not 16	897,273
18	conference abstract.pt.	2,614,376
19	17 not 18 [NHS-EED filter for Embase]	764,887
20	random:.tw.	1,229,844
21	clinical trial:.mp.	1,480,008
22	exp health care quality/	2,545,178
23	or/20–22 [McMaster Embase sensitive therapy filter]	4,306,191
24	exp urine incontinence/	67,410
25	(((urin* or bladder) adj3 incontin*) or encopresis or enures* or ((urin* or bladder) adj2 control) or (urin* adj2 (leak* or hold*)) or icq or interstim).ab,kw,ti.	53,445
26	24 or 25 [urinary incontinence]	80,281
27	19 and 23 and 26	1,343
28	(NCT02316275 or ISRCTN57746448 or NTR3783 or NCT01239836 or NTR1871 or NCT00814749 or NTR1248 or NCT00611026 or NTR1181 or NCT00509730 or NCT00498888 or NCT00444925 or NCT00425100 or ACTRN12605000755639 or NCT00200031 or ISRCTN97769568).ab,kw,ti.	8
29	(MiniMo or VUSIS or “Value of Urodynamics Prior to Stress Incontinence Surgery”).ab,kw,ti.	49
30	28 or 29 [relevant urinary incontinence trials]	56
31	27 or 30	1,398
32	remove duplicates from 31	1,367

**Table 3:** Bibliographic, clinical, and methodological characteristics of studies included in this review

Reference	Publication year	Country	Price year	Currency	Sample size	Setting	Interventions compared	Cost analysis	Costing transferability	Costing method
Albers-Heitner et al. [33]	2012	Netherlands	2007	Euros	384	General practice	Care as usual by GPs versus care by nurse specialist	CUA	B	I
Ankardal et al. [14]	2007	Sweden	2003	Euros	714	Hospital	Open Burch colposuspension, laparoscopic colposuspension and TVT	CEA	A	I
Boyers et al. [15]	2013	UK	2011	Pounds	137	Secondary care	SIMS (Ajust) versus SMUS (TVT-O)	CUA	A	I
Brunenberg et al. [16]	2006	Netherlands	2002	Euros	1000*	General practice	Duloxetine versus PFMT	CEA	A	I
Dumville et al. [17]	2006	UK	2002–2003	Pounds	286	Gynecological surgical centers	Open colposuspension versus laparoscopic colposuspension	CUA	A	I
Foote et al. [35]	2007	Australia	1995	Australian dollars	205	Pelvic floor unit, hospital	Nurse continence advisor versus urogynecologist	CUA	B	I
Hana et al. [18]	2012	Bosnia	*2007–2010/2011	Euros	60	Hospital	Vaginoplasty versus TVT-O	CBA	A	I
Jacklin et al. [34]	2010	UK	2007–2008	Pounds		Hospital	TVT versus duloxetine	CUA	B	I
Kilonzo et al. [19]	2004	Ireland	2001	Pounds	?	Hospital	TVT versus other surgical procedures	CUA	A	I
Kobelt and Fianu-Jonasson [20]	2006	France and Sweden	2004	Euros	159	Hospital	NASHA/Dx gel versus TVT	CUA	A	I
Kondo et al. [38]	2006	Japan	2005	US dollars	60	Hospital	TVT or pubovaginal sling	Cost comparison	D	IV
Kumar et al. [49]	2017	USA	1998	US dollars	–	Hospital	Sling versus Burch	CUA	D	III
Kung et al. [21]	1996	Canada	1994	Canadian dollars	62	Tertiary referral practice	Laparoscopic versus abdominal Burch	CEA	A	I
Kunkle et al. [39]	2015	USA	2013	US dollars	**–**	Hospital	BA versus MUS	CUA	D	IV
Lamb et al. [22]	2009	UK	2004–2005	Pounds	174	Primary care	Group or individual physiotherapy sessions	Cost comparison	A	I
Lier et al. [44]	2011	Canada	2007	Canadian dollars	194	Hospital	TOT versus TVT	CUA	C	I
Lier et al. [45]	2016	Canada	2011	Canadian dollars	195	Hospital	TOT versus TVT	CUA	C	I
Lo et al. [23]	2013	Canada	2010	Canadian dollars	18	Hospital	Burch colposuspension, laparoscopic two-team sling and TOT	Cost comparison	A	I
Maher et al. [40]	2005	Australia	1998	Australian dollars	45	Tertiary referral practice	Pubovaginal sling versus transurethral Macroplastique	Cost-outcome description	D	IV
Manca et al. [24]	2003	UK	*1999–2000	Pounds	214	Hospital	TVT versus colposuspension	CUA	A	I
Mihaylova et al. [25]	2010	UK	2007	Pounds	1510	Primary care	Duloxetine, duloxetine + nonsurgical conservative treatment, nonsurgical conservative treatment	CUA	A	I
Montesino-Semper et al. [36]	2013	Spain		Euros	69	Primary care and hospital	Surgical intervention (sling/mesh) versus therapeutic abstention	Cost comparison	B	I
Moore et al. [26]	2003	Australia	1998	Australian dollars	110	Tertiary referral center	Nurse continence advisor therapy versus standard urogynecologist care	Cost-outcome description	A	I
Norton et al. [41]	2016	USA	2014	US dollars	630	Tertiary referral practice	OE versus OE plus UDS	Cost comparison	D	IV
Persson et al. [27]	2002	Sweden	1998?	Euros	79	Hospital	Laparoscopic colposuspension versus TVT	Cost comparison	A	I
Ramsay et al. [37]	1996	UK	1995	Pounds	74	Hospital	Inpatient versus outpatient continence program	Cost-outcome description	B	I
Richardson et al. [46]	2013	USA	2010	US dollars	Model	Hospital	MUS versus abdominal sacrocolpopexy	CUA	C	IV
Richardson et al. [47]	2014	USA	2012	US dollars	Model	Primary care and hospital	Conservative therapy (pessary or PFMT) versus MUS	CUA	C	IV
Sand et al. [48]	2013	USA	2012	US dollars	Model	Hospital	Cost effectiveness of five strategies for SUI patients in whom conservative treatment failed	Cost comparison	C	IV
Seklehner et al. [43]	2014	USA	2012	US dollars	Model	Tertiary referral practice	Retropubic MUS versus TO MUS	CUA	B	IV
Sjӧstrӧm et al. [28]	2015	Sweden	2010	Euros	250	Primary care	Two treatment programs (internet-based treatment program versus post) and no treatment	CUA	A	I
Sjӧstrӧm et al. [29]	2017	Sweden	2013	Euros	123	Primary care	Mobile app for treatment of SUI	CUA	A	I
Subak et al. [30]	2014	USA	2012	Dollars	491	Hospital	Burch versus fascial sling surgery	Cost comparison	A	I
Valpas et al. [31]	2006	Finland	2000	Euros	121	Hospital	TVT versus laparoscopic mesh colposuspension	CEA	A	I
Vermeulen et al. [32]	2016	Netherlands	2010	Euros	350	General practice	Pro-active approach of diagnostics and treatment versus comparison	CUA	A	I
Von Bargen and Patterson [42]	2015	USA	2012	Dollars	Model	Primary care and hospital	Nonsurgical versus surgical treatments	CUA	D	IV

*CUA* cost–utility analysis, *CEA* cost-effectiveness analysis

### Data extraction

Included studies reported a range of cost estimates, covering various health care activities relevant for diagnosis and treatment of SUI, with varying levels of aggregation or detail. Data were extracted and entered into an Excel sheet.

### Costing transferability and methodology level

Table 4 shows the costing transferability and costing methodology of the articles included. As presented, most included articles score an A-I level on transferability and methodology [14–32]. This means that micro- or quasi-micro costing was applied and that all components of costs were described. The second largest categories were B-I and D-IV, in which use of relative units with all components and charge data with the scope of costing was given respectively. B-I means that relative data for all components have been given [33–37]. D-IV generally means that unmodified charge data for the entire procedure was given [38–42]. All other categories (i.e., BIV, CI, CIV, DIII) applied to only three of the included articles or fewer. Least represented categories were B-IV [43], C-I [44, 45], C-IV [46–48], and D-III [49].

**Table 4:** Costing transferability and costing methodology [7, 8]

Costing methodology	I	II	III	IV	V
Costing transferability					
A	Ankardalet al. [14] Boyers et al. [15] Brunenberg et al. [16] Dumville et al. [17] Hana et al. [18] Kilonzo et al. [19 Kobelt and Fianu-Jonasson [20 Kung et al. [21] Lamb et al. [22] Lo et al. [23 Manca et al. [24] Mihaylova et al. [25] Moore et al. [26] Persson et al. [27] Sjӧstrӧm et al. [28] Sjӧstrӧm et al. [29] Subak et al. [30] Valpas et al. [31] Vermeulen et al. [32				
B	Albers-Heitner et al. [33] Jacklin et al. [34 Foote et al. [35] Montesino-Semper et al. [36] Ramsay et al. [37]			Seklehner et al. [43]	
C	Lier et al. [44] Lier et al. [45]			Richardson et al. [46 Richardson et al. [47 Sand et al. [48]	
D			Kumar et al. [49]	Kondo et al. [38] Kunkle et al. [39] Maher et al. [40] Norton et al. [41 von Bargen and Patterson [42]	

### Exploration of reported cost estimates

The costs of hospitalization in different countries and clinical departments were investigated.

**Fig. 2:**
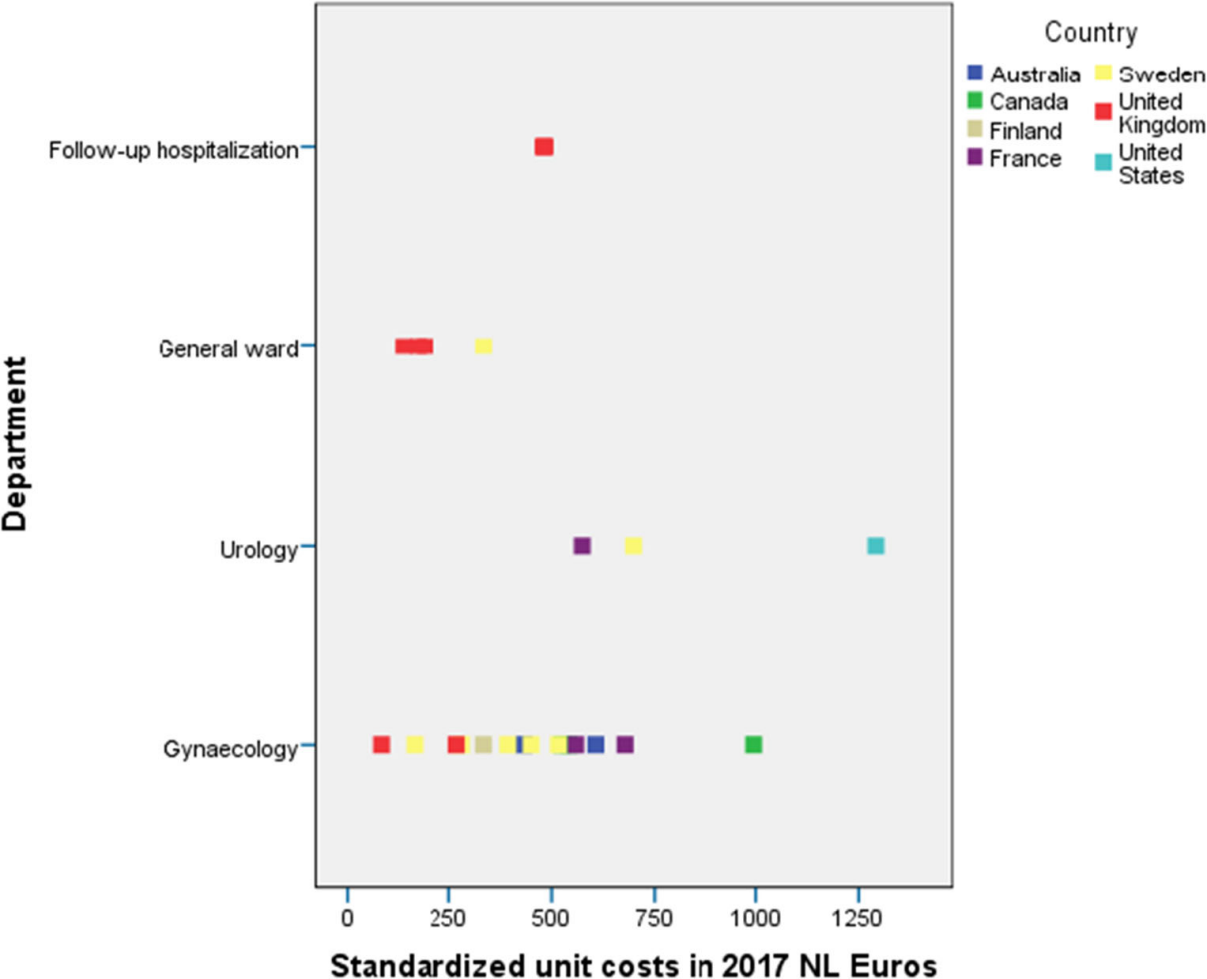
Standardized unit costs per day of hospitalization vary between and within countries

**Fig. 3:**
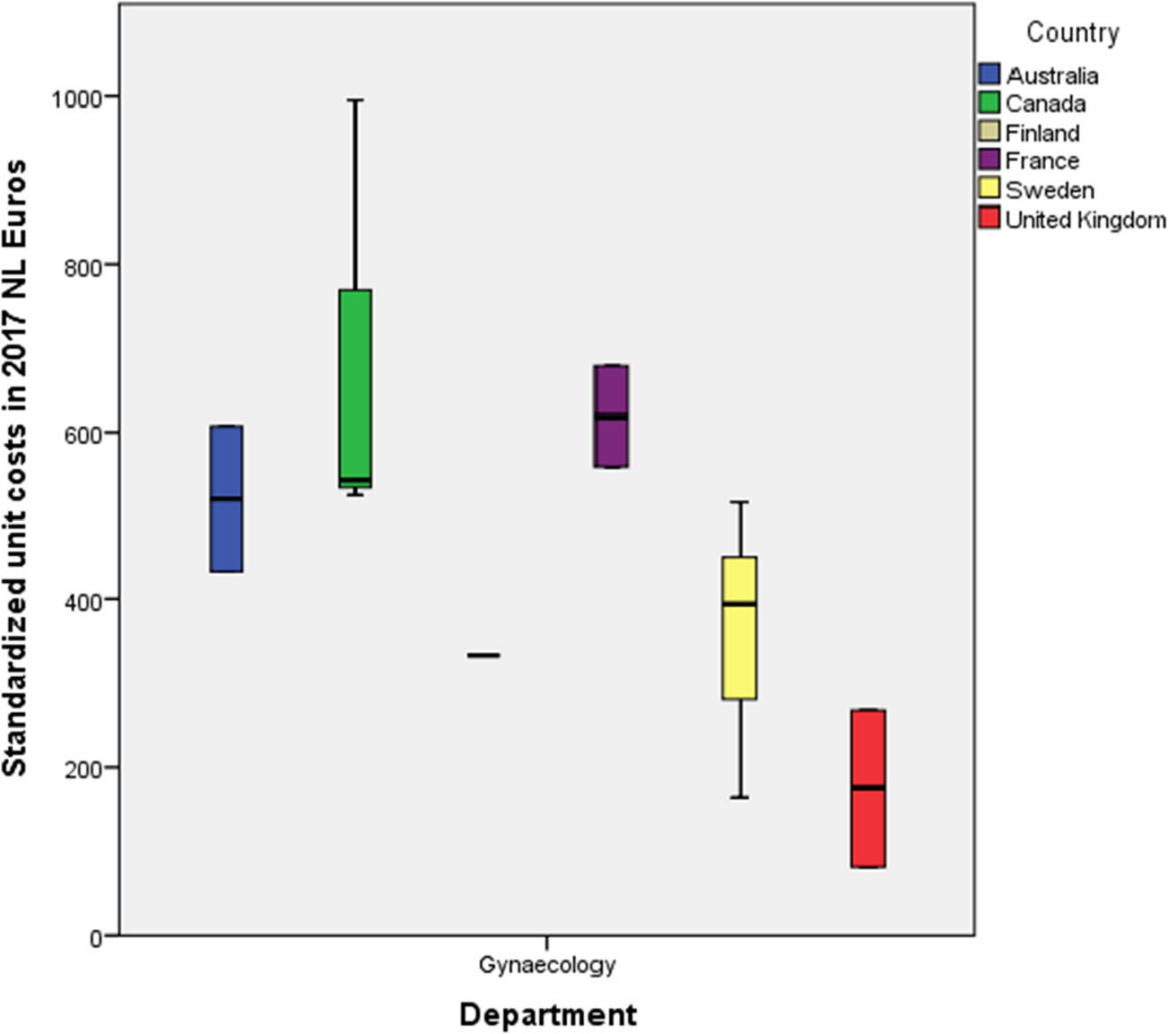
Standardized unit costs per day of hospitalization day in the gynecology department vary between countries

Standardized unit costs per day of hospital stay varied considerably within and between countries (Fig. 2). Overall, unit costs for hospital admissions in gynecology ranged between 82 and 1292 Euros per day. The lowest estimates were reported for the UK, where prices varied between 82 and 518 Euros (Fig. 3) [15, 17, 22, 24, 37]; whereas the highest estimates were observed in the USA, namely 1,292 Euros per day [43]. Hospitalization in a general ward was less expensive than in a urology department (median 208 versus 855 Euros) [15, 17, 20, 24, 43]. Costs reported for staying in a gynecology department ranged from 82 Euros per day to 995 Euros per day [14, 35, 20, 23–24, 40, 27, 37, 31]. Costs that were used for the descriptive analyses were derived from the studies included. These studies either used national sources [14, 15, 17, 20, 22, 23–24, 27, 37, 40], or unknown sources [31, 35, 37, 43].

Costs of gynecological consultation range from 30 Euros in France to 228 Euros per hour in the UK [20, 24]. The cost of a TVT device ranges from 431 Euros in Finland to 994 Euros in Canada [31, 44, 45]. TVT surgery per minute costs range from 25 Euros in Finland to 82 Euros in Sweden [27, 31]. Total costs for TVT range from 1,224 Euros in Ireland to 5,809 Euros for inpatient care in France [19, 20].

Different units to express the costs of hospital stay were identified, including average cost per day [14, 17, 20, 24, 27, 31, 35, 37, 40, 43] or NHS cost day [22]. Occasionally, cost per hour or cost per night was given [15, 23, 31, 43]. As none of these articles described the duration of a hospital day, we could not convert cost per hour/night to the more common unit of a full hospital admission day.

**Fig. 4:**
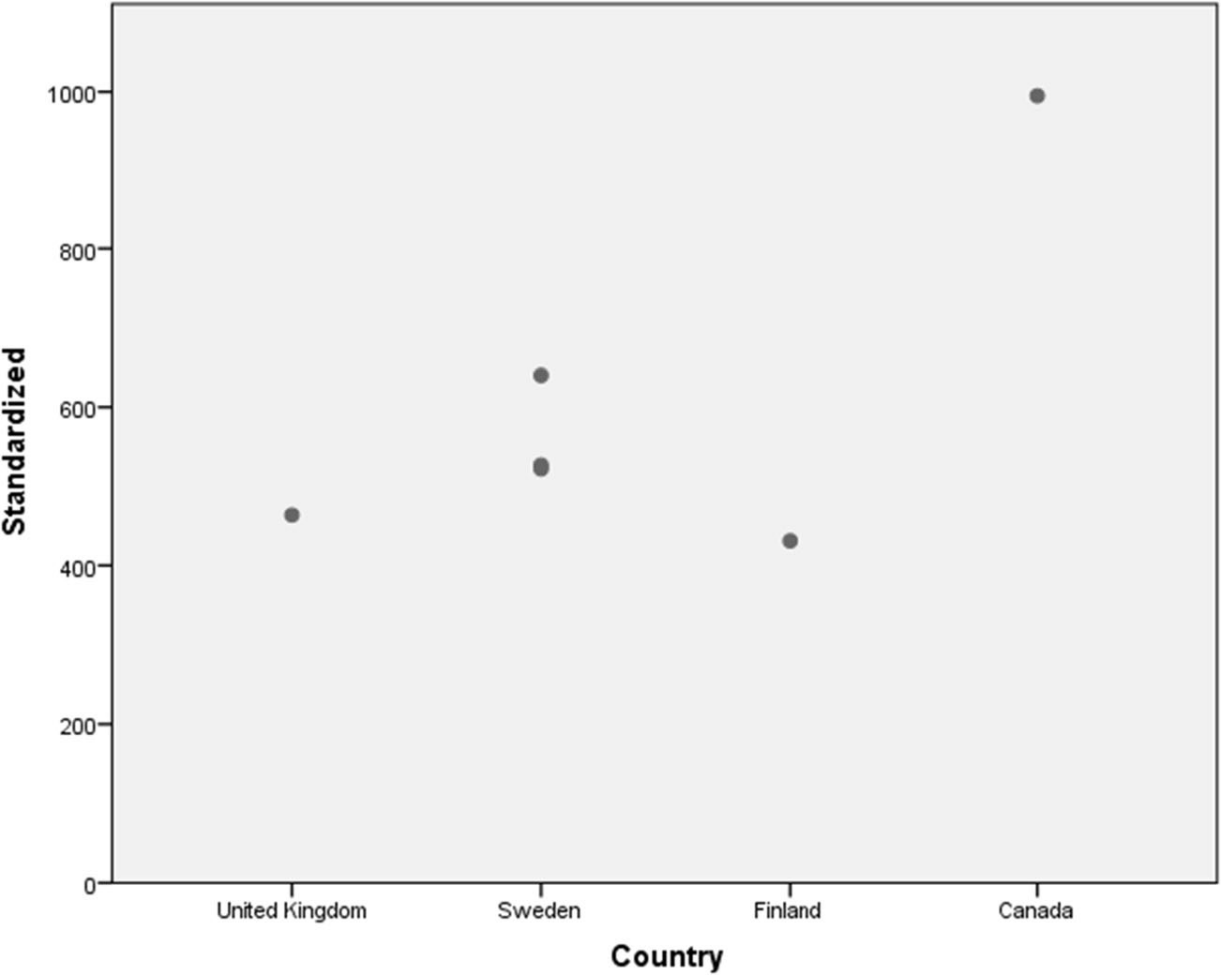
Standardized unit costs for tension-free vaginal tape equipment vary between countries

Figure 4 shows the results of the TVT material given. Costs from the UK, Sweden and Finland were comparable, but costs from Canada were significantly higher.

**Fig. 5:**
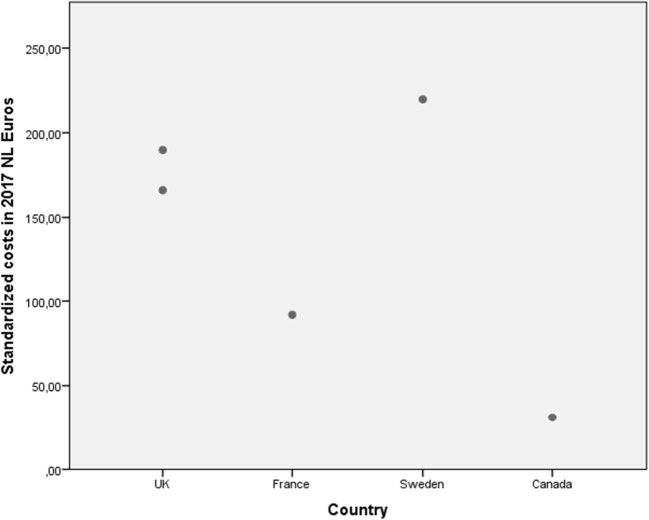
Standardized unit costs for urodynamic testing vary among countries

Figure 5 shows the costs of urodynamic testing. Costs in Canada are significantly lower than in the UK, Sweden, and France.

## Discussion

We have assessed the variation in unit cost estimates that are reported in economic evaluation studies and have provided an overview of cost estimates for different components in the diagnosis, treatment, and follow-up of SUI. Our study suggests that for many commonly reported cost units in the field of SUI, the cost estimates vary widely among studies and among countries. In addition, reported costs are not commonly listed in detail. Our study provides evidence that variability in cost estimates results from differences in interventions and health care services among countries, and that sources used to derive costs and the way in which units are defined cause dissimilarities in costs.

### Strengths and limitations

Both a strength and limitation of our study is the focus on the clinical area of SUI. A clear clinical focus limits the range of interventions and health care services provided for this condition. On the other hand, this focus may limit generalizability, as we do not know whether such heterogeneity in cost estimates would be similar in other fields of medicine. Nevertheless, the costs of hospital admissions for instance, one of our main outcomes, is not necessarily based on the corresponding intervention.

Second, the classification criteria we used to define costing methodology and transferability could be debated. We applied the criteria used by Fukuda et al., which makes our methods consistent with earlier work [8]. As these criteria are not very strictly defined and may be applied differently to cost components within a single study, this classification approach may not be optimal. On the other hand, a common framework to characterize and summarize studies in terms of costing methodology and/or transferability is currently lacking. The criteria of Fukuda et al. could contribute to such a framework.

Finally, we did not combine cost estimates to a pooled average. To our knowledge, few health economic studies in the field of gynecology have been published that systematically review cost estimates produced by health economic evaluation research [50]. Therefore, there is no evidence that studies are consistent at reporting transparently, and transferable cost estimates are scarcely used. It is unclear whether unit costs within countries can be pooled to a national average cost estimate, and to what extent unit cost estimates can be generalized to other countries [51, 52]. Oppong et al. conducted a systematic review to evaluate health economic studies that were performed in multinational trials. The authors concluded that pooling of the outcomes was impossible because, for instance, studies did not use cost prices from all countries that participated in the specific trial [53]. These findings underline the need for better pooling strategies when using cost estimates and unit cost estimates in clinical trials for each country enrolling patients in the trial. Given the large variety of reported cost estimates and different cost estimates or cost units, our data were not homogeneous enough to allow pooling. Therefore, this review has resulted into a more descriptive evaluation of the study results instead of providing pooled estimates for different levels of aggregation that are not necessarily directly linked to a specific intervention. In 1998, Schulman et al. presented a method to use cost estimates to calculate relative medical cost indices that could be used in cost estimates for multinational purposes [54]. However, to allow generalization to similar settings or transfer to other countries, reported costs that are included in the model need to be consistent [51, 52]. Thus far, there is no best practice for reporting generalizable and transparent costs. As a guideline, the use of costing methodology and costing transferability as described in the methods of this article incorporating the definitions of Fukuda et al. should be utilized by researchers when establishing cost data [8].

### Transparent definition of costing units of health care use

The way in which units of resource use are currently reported introduces bias that can have a negative effect on the interpretation of costs from research studies. Although most of our included studies scored high in transferability, we did see that the taxonomy in definitions of units was often not transparent. For instance, although most articles describe hospital stay as mean costs per day, this is not particularized any further. For instance, hospital stay was defined as average cost per day [14, 17, 20, 24, 27, 31, 35, 37, 40, 43] or NHS cost per day [22]. This terminology does not reveal what the cost units actually entail as no further description of a day has been given. Consequently, it is not clear whether all costs that have been included in Figs. 2 and 3 are actually comparable. In these figures, no distinction is made between inpatient and outpatient care, type of anesthesia, type of treatment, and treatment duration. Therefore, the component hospital stay could vary depending on its source.

Moreno and Montesino have described the economic impact of an inpatient versus outpatient treatment of SUI [11]. These authors describe all components of a hospital stay, including personnel costs, materials, medicines, laundry, etc. These specific costs are not generally mentioned in our included articles. Some articles do describe the separate units and most mention “hospital bed” as the unit to be costed, but it is unclear whether all separate units include the same components. The NHS calculates the cost of a hospital bed according to the treatments that are required for the average patient occupying that bed [55]. In an editorial, Bryce Travers explains that the daily cost of a hospital bed depends on what support a particular patient needs in the patient’s specific care pathway [56]. However, this would make costing personalized and therefore perhaps more complex than previously thought. But, a guide such as that given by Moreno and Montesino that comprehensively explains different costing units in the diagnosis and treatment of SUI would improve transparency of costs and transferability of costs between settings and countries [11].

### Differences between countries

Standardized unit costs vary within and among countries. These differences in costs do not seem to be consistent and from our limited data we cannot determine whether the differences we observe are statistically relevant. What we do see is that variation within countries can be the result of reporting absolute versus relative unit cost estimates or the use of varying units for consultation costs, for instance, costs per hour, costs per visit, or costs per consultation. Moreover, outpatient and inpatient costs are both reported and also depend on whether the patient is admitted to a ward or gynecology department. Overall, we have not been able to identify a noteworthy pattern in the variation of unit cost estimates.

### Implications: a recommendation for future economic evaluation studies

#### Sources

Most studies use national guidelines as a source for the price of the cost estimates. However, we also found studies that obtained the unit cost estimates by interviewing doctors, surveying hospitals or approximating costs from charges. Using different sources for deriving unit cost estimates is not necessarily wrong; Fukuda et al. describe that some costing methodologies are more accurate than others [8]. However, national cost estimates and local charge data are not the same. Therefore, it should be the aim of researchers to use the highest possible source of estimates to produce meaningful results and to draw transparent and transferable conclusions. The best available evidence should be used for clinical decision-making [57]. When costs are not based on the best available evidence, costs are not reliable. Therefore, as should be done when deriving evidence for the efficacy and safety of a treatment, deriving evidence on costs should also be done with great care.

#### Transferability and costing method

As a consequence of heterogeneity in studies reporting unit measures with associated costs, it is not possible to draw immediate conclusions with regard to the cost-effectiveness of new treatments. Especially when new treatments are studied, reported costs should entail all included unit measures and not only overall cost differences. Only then could the cost-effectiveness of the new treatment option—with care—be transferred and compared with the cost-effectiveness of other interventions for SUI. For accurate calculation of pooled cost estimates that are based on multiple evidence sources, the results of these sources need to be presented in a transparent and reproducible way [9, 58–59].

#### Transfer between countries

In this review, differences among countries are accounted for using transparent methods to adjust cost estimates. Variation in economic estimates that are attributable to differences between countries seems of low significance [58], but study outcomes are not generalizable when economic circumstances and differences in health systems across countries are not taken into account [53]. In 1998, Schulman et al. presented a way of establishing cost estimates, “relative medical cost indices,” that could be used as a method to transfer costs from one country to another [54]. Such a method can unfortunately not be universally used throughout time: the model is susceptible to differences in discounting among countries, and the indices are therefore not fixed. Oppong et al. has given more information about differences among countries and how these affect generalizability. Oppong et al. propose that overcoming systematic differences due to economic circumstances and health systems and improving generalizability can be achieved by:

1. Carefully selecting countries for inclusion in studies
2. Using a checklist to overcome heterogeneity
3. Use protocols on treatment patterns
4. Reporting costs from different perspectives

Additionally, in the ISPOR recommendations from 2009, Drummond et al. suggest models that might be used to correct for differences among countries [59].

Specific methodology has been used in our review to approximate differences between economic circumstances; however, this does not automatically accommodate differences in healthcare systems.

## Conclusion

To facilitate insight into the variation of costs we presented an overview of a commonly used unit—hospital admission—and reported corresponding cost estimates. We also described the source of these costs and the way in which the cost estimate was calculated. Heterogeneity was observed in unit costs for most units; at both a more aggregated level (for instance a surgical procedure) and for units at a lower level of aggregation (for instance hourly wages for nurses or medical specialists). Heterogeneity in cost estimates is likely the consequence of sources used, actual cost differences among countries, lacking transparency in costing procedures or time-related factors [7, 8]. Ultimately, the results of our study imply that every research study that includes costs has to ascertain that the reported costs are valid and reliable. Consequently, these costs should be used with caution in cost-effectiveness studies.

Studies used varying taxonomy and definitions for estimated costs, and unit costs vary considerably between settings and countries. To minimize variation in unit costs, more uniform taxonomy definitions of units and cost analyses are required. Only then are cost estimates comparable or even transferable among countries and can be used in meta-analyses of cost-effectiveness studies.

Available cost-effectiveness outcomes should be interpreted with care, as reported cost estimates can be outdated, biased or unreliable. The methodology of economic evaluation research would benefit from quality standards as proposed in this review. Such quality standards are aimed reducing methodological heterogeneity and allow exploration and explanation of clinical heterogeneity in cost estimates. Available cost-effectiveness results are likely most valid for (or even limited to) particular health care contexts; more standardized methods, taxonomy, and definitions will enhance transferability to other contexts.

## Abbreviations

CEA: Cost-effectiveness analysis
SUI: Stress urinary incontinence
UI: Urinary incontinence
